# Bone Reporting and Data System on MRI (Bone-RADS-MRI): a validation study by four readers on 275 cases from three local and two public databases

**DOI:** 10.1186/s13244-025-02040-3

**Published:** 2025-07-17

**Authors:** Yue Xing, Yangfan Hu, Xianwei Liu, Defang Ding, Shun Dai, Liangjing Lyu, Guangcheng Zhang, Shiqi Mao, Qian Yin, Junjie Lu, Jiarui Yang, Yang Song, Huan Zhang, Chengzhou Li, Weiwu Yao, Jingyu Zhong

**Affiliations:** 1 Department of Imaging, Tongren Hospital, Shanghai Jiao Tong University School of Medicine, Shanghai, 200336 China; 2 Shanghai Key Laboratory of Flexible Medical Robotics, Tongren Hospital, Institute of Medical Robotics, Shanghai Jiao Tong University, Shanghai, 200336 China; 3 Department of Orthopedics, Shanghai Sixth People’s Hospital, Shanghai Jiao Tong University School of Medicine, Shanghai, 200233 China; 4 Department of Medical Oncology, Shanghai Pulmonary Hospital, Tongji University School of Medicine, Shanghai, 200433 China; 5 Department of Pathology, Renhe Hospital, Shanghai, 200431 China; 6 Department of Epidemiology and Population Health, Stanford University School of Medicine, Stanford, CA 94305 USA; 7 Department of Biomedical Engineering, Boston University, Boston, MA 02215 USA; 8 MR Research Collaboration Team, Siemens Healthineers Ltd., Shanghai, 200126 China; 9 Department of Radiology, Ruijin Hospital, Shanghai Jiao Tong University School of Medicine, Shanghai, 200025 China; 10 Department of Nuclear Medicine, Tongren Hospital, Shanghai Jiao Tong University School of Medicine, Shanghai, 200336 China

**Keywords:** Bone neoplasms, Clinical decision-making, Reproducibility of results, Magnetic resonance imaging

## Abstract

**Objective:**

To evaluate the reproducibility and effectiveness of the Bone Reporting and Data System on MRI (Bone‐RADS-MRI) for incidental solitary bone lesions in adults.

**Materials and methods:**

We retrospectively included 275 MRI cases from three local and two public databases, respectively. All the cases were histopathologically or clinically confirmed bone lesions, or “do not touch” lesions with typical appearance and remained stable for at least two years. Each lesion with gender, age, and clinical history was categorized according to the Bone-RADS algorithm by two musculoskeletal radiologists and two non-musculoskeletal radiologists. The Bone-RADS categories were as follows: Bone-RADS-1, likely benign, leave alone; Bone-RADS-2, incompletely assessed on imaging, perform different imaging modality; Bone-RADS-3, intermediate, perform follow-up imaging; Bone-RADS-4, suspicious for malignancy or need for treatment, biopsy and/or oncologic referral. Inter-reader agreement was evaluated. The diagnostic performance of the Bone-RADS-MRI was measured for distinguishing intermediate or malignant lesions or osteomyelitis from benign lesions. The histopathology results, clinical diagnosis, or follow-up were used as a standard reference.

**Results:**

There were 165 intermediate or malignant lesions or osteomyelitis, and 110 benign lesions, respectively. The inter-reader agreements between two musculoskeletal and between two non-musculoskeletal radiologists were both moderate (weighted kappa 0.572 and 0.520). The diagnostic performance for identifying intermediate or malignant lesions or osteomyelitis ranged according to radiologists with sensitivities of 88.5% to 94.5%, specificities of 55.5% to 74.5%, and accuracies of 76.4% to 82.9%.

**Conclusion:**

Bone-RADS-MRI is effective for identifying bone lesions that need further treatment, but it has only moderate reliability for readers with different specialties and experience.

**Critical relevance statement:**

With local and public databases, Bone-RADS-MRI has been demonstrated to be a reliable algorithm for musculoskeletal and non-musculoskeletal radiologists with varying experience and an effective tool for identifying incidental solitary bone lesions that “need treatment” in adults.

**Key Points:**

- Bone-RADS-MRI needs clinical validation for inter-reader agreement and diagnostic performance.
- Bone-RADS-MRI achieved moderate agreements between musculoskeletal and non-musculoskeletal radiologists, respectively.
- Bone-RADS-MRI presented high sensitivities but low specificities for identifying “need-for-treatment” bone lesions.

**Graphical Abstract:**

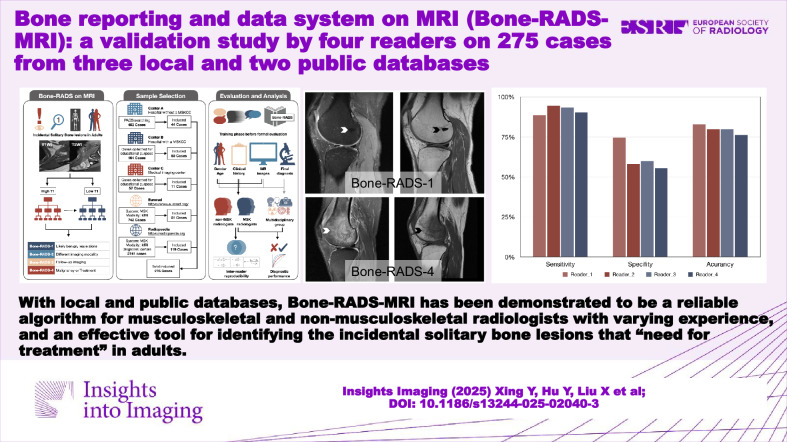

## Introduction

Bone lesions are more frequently detected with the increasing number of radiological examinations [1]. However, the diagnosis of bone lesions can be difficult due to the broad spectrum of differential diagnoses and overlapping imaging features [2–6]. Further, with a limited number of cases [7, 8], radiologists may not have enough experience to develop the ability to diagnose bone lesions. Malignant bone tumors are related to poor prognoses and should be identified as soon as possible; while some are not touchable, others may only need following [9, 10]. There is a need for multidisciplinary communication on bone lesions [11], but the consensus on systematic and standardized approaches for bone lesion evaluation is desired [12]. The clinical presentations of bone tumors are usually nonspecific, such as pain, discomfort, and mass. Radiologists, pathologists, and clinicians should collaborate to reach an accurate diagnosis for appropriate treatment selection. For example, dedifferentiated chondrosarcomas may be considered as benign tumors if the biopsy sample contains only benign components, whereas radiologically aggressive features can suggest their malignancy [13]. New bone formation in giant cell-rich osteosarcoma is not always visible on radiological examinations, but pathologists can find evidence of malignancy, including osteoid formation and multinucleated giant cells under the microscope [14].

Following the success of breast imaging report and data system (BI-RADS) [15], a series of RADS have been proposed, including colon, liver, lung, head and neck, ovarian-adnexal, prostate, thyroid, etc. [16]. The RADS allows the structured reporting and sharing concepts, to enhance communication and collaboration among health professionals from different subspecies [17, 18]. Currently, several RADS for bone lesions have been proposed [17]. Radiological Evaluation Score for Bone Tumors (REST) on radiograph was developed using a cohort of 100 patients with primary bone tumors [19]. Bone-RADS on radiography for tumor risk stratification and management was established in consensus by expert opinions of the Committee of the American Congress of Radiology (ACR) [20]. The ACR Bone-RADS has been validated in two retrospective studies and showed high sensitivity and moderate inter-reader agreement [21, 22]. The system Bone tumor imaging RADS (BTI-RADS) was developed with 230 patients with histologically confirmed bone tumors, but required both CT and MRI for evaluation [23]. Osseous tumor RADS (OT-RADS) was built using MRI scans from 136 patients with bone tumors [24], and further validated within 133 patients with extra DWI images [25]. Bone-RADS for solitary bone lesions on CT or MRI was proposed by the Society of Skeletal Radiology (SSR) according to consensus of twelve musculoskeletal radiologists and one orthopedic oncologist [26]. These systems were expected to improve the assessment and subsequent management of bone lesions.

The SSR Bone-RADS on CT and MRI includes four algorithms for CT lucent lesions, CT sclerotic/mixed lesions, MRI T1 high signal lesions, and MRI T1 low signal lesions, respectively [26]. The readers can assess bone lesions following the algorithms according to the presence or absence of pain, malignancy history, and imaging characteristics. Bone-RADS is presented in a decision tree approach in order to simplify its usage. It mainly depends on the imaging characteristics; thus, it is usable if the clinical history is incomplete. It is developed for both CT and MRI, whether they are contrast-enhanced or not, and has the same categories on these two modalities that improve the understanding and interpretation. However, it is of importance to note that only Bone-RADS-CT has been validated [27–30]. Considering the important role of MRI in bone tumor assessment [31–33], it is necessary to validate the inter‐reader agreement and diagnostic performance of Bone-RADS-MRI.

Therefore, this study aimed to evaluate the reproducibility and effectiveness of the Bone‐RADS-MRI for incidental solitary bone lesions in adults by four readers using three local databases and two public databases.

## Materials and methods

### Study design

**Fig. 1:**
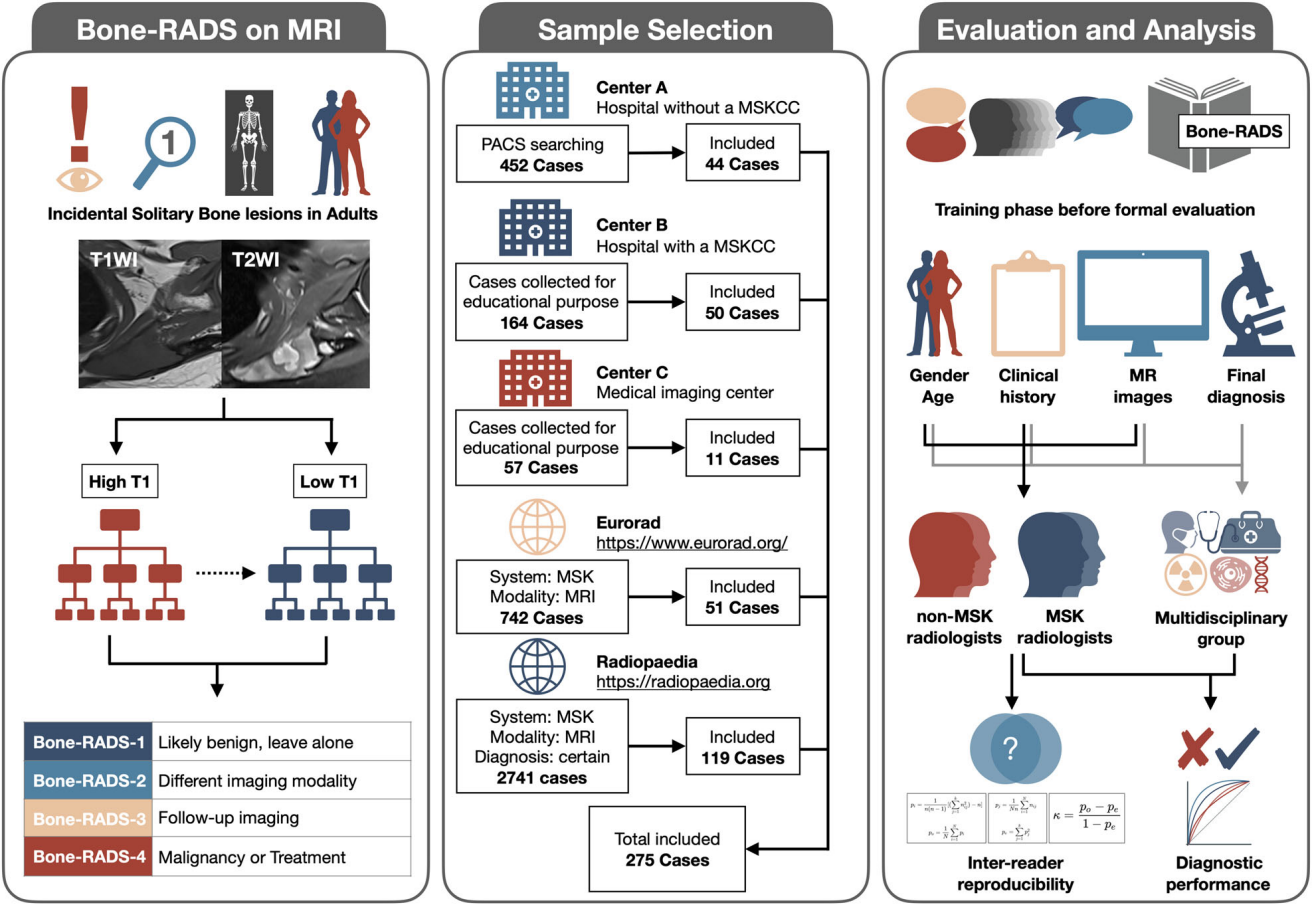
Bone-RADS-MRI and workflow of the study. Bone-RADS, Bone Reporting and Data System; MSKCC, musculoskeletal cancer center; PACS, picture archiving and communication system

This retrospective study was conducted using cases from three local institutional databases and two public databases (Fig. 1). The study has been approved by the institutional review board, and the written informed consents from participants were waived. The two public databases, Eurorad (https://www.eurorad.org) and Radiopaedia (https://radiopaedia.org), were under the Creative Commons License CC BY-NC-SA 4.0 and CC BY-NC-SA 3.0, respectively. We have obtained the permission for appropriate use of the cases from these two databases.

### Study sample selection

The sample selection was conducted by two musculoskeletal radiologists with 6 and 7 years of experience (J.Y.Z. and Y.X.), respectively [23–27]. The cases should fulfill the following criteria: (a) >= 18 years old; (b) solitary bone lesions; (c) complete MRI examination, defined as the MRI examination at least including T1-weighted images without fat suppress, and T2-weighted images with/without fat suppress; (d) clinical history available; and (e) histologically or clinically confirmed diagnosis, or those with typical appearance of “do not touch” lesions and remain stable for at least two years. The following cases were excluded: (a) overlapping examinations of the same patient; (b) follow-up examinations of the same case; (c) examinations after treatment; (d) unsatisfactory image quality; (e) allowed for non-commercial use.

The picture archiving and communication system of center A was searched by using terms related to bone lesions, and the eligibility of 452 potential cases between 01 Jan 2019 and 30 Jun 2023 was evaluated. The 183 potential cases confirmed by histology between 01 Jan 2018 and 30 Jun 2020 from center B were collected for educational purposes and assessed. The 56 potential cases confirmed by histology between 01 Jan 2010 and 31 Dec 2015 from center C were collected for research purposes and assessed. The 742 potential cases until 30 Jun 2024 from Eurorad were identified by using the advanced search of the musculoskeletal system and imaging modality of MRI, and then their eligibility was estimated. The 2741 potential cases until 30 Jun 2024 from Radiopaedia were distinguished by using the filter of musculoskeletal system, imaging modality of MRI, and ‘certain diagnosis’, and then their eligibility was estimated. The patient demographics, clinical history, MRI images, and final diagnosis were collected. Finally, 275 cases were included, in which 44, 50, 11, 51, and 119 cases were from center A, center B, center C, Eurorad, and Radiopeadia databases, respectively. The detailed sample selection process is presented in Supplementary Note S1.

### Final diagnosis and Bone-RADS

The final diagnosis of cases and reference standard of Bone-RADS for diagnostic performance evaluation was established by the multidisciplinary group consisting of six radiologists (J.Y.Z., Y.X., L.J.L., C.Z.L., H.Z., and W.W.Y.), one orthopedist (G.C.Z.), one oncologist (S.Q.M.), and one pathologist (Q.Y.). The final diagnosis was reached by the multidisciplinary group according to the histological or clinical confirmation, typical appearance of “do not touch” lesions and follow-ups for at least two years [23–27]. We did not establish the reference standard for Bone-RADS-2 or Bone-RADS-3 lesions, since its suitability may change according to the experience and opinion of readers, as well as the attitude and socioeconomic status of the patients. For the evaluation of diagnostic performance, the intermediate or malignant lesions, or osteomyelitis, were defined as positive cases, and benign lesions were defined as negative cases. We defined the results of rating as follows, true positive (TP), positive cases that diagnosed as Bone-RADS-2, 3, or 4; false positive (FP), negative cases that diagnosed as Bone-RADS-2, 3, or 4; false negative (FN), positive cases that diagnosed as Bone-RADS-1; and true negative (TN), negative cases that diagnosed as Bone-RADS-1. These definitions allow us to tell whether the Bone-RADS is effective for certainly confirming that lesions designated Bone-RADS-1 are truly benign processes that require no additional work-up; meanwhile, it is effective for not ignoring Bone-RADS-4 lesions that are spacious for malignancy or need for treatment. The detailed methodology for the establishment of the final diagnosis and reference standard of Bone-RADS is presented in Supplementary Note S2.

### Bone-RADS training and evaluation

Two musculoskeletal radiologists with 6 and 7 years of experience (J.Y.Z. and Y.X.) created anonymized image sets in a PowerPoint format (Office 365; Microsoft) and prepared their gender, age, and clinical history in an Excel format (Office 365; Microsoft). This method was considered to be effective for validation of a RADS in the musculoskeletal system [34]. These documents with images and patient information of each lesion were evaluated by four readers, including two musculoskeletal radiologists with 6 and 8 years of experience (Y.F.H. and D.F.D.), and two non-musculoskeletal radiologists, both with 9 years of experience (S.D. and X.W.L.), respectively. Before the formal assessment, the readers studied the Bone-RADS algorithm document and tested their comprehension of the document by using ten attached representative cases [26]. The readers were told to rate the Bone-RADS categories for each lesion according to following criteria: Bone-RADS-1, likely benign, leave alone; Bone-RADS-2, incomplete assessed on imaging, perform different imaging modality; Bone-RADS-3, intermediate, perform follow-up imaging; Bone-RADS-4, suspicious for malignancy or need for treatment, biopsy and/or oncologic referral. During formal evaluation, the PowerPoint document of images and the Excel document of patient information were provided to the readers, but the source of the case, final diagnosis, and databases were blinded. The cases were randomly presented to the readers, and there were no time limits for the rating process of each case. All the readers finished the Bone-RADS rating within a week. The detailed methodology for training and formal evaluation is presented in Supplementary Note S3.

### Statistical analysis

The statistical analysis was conducted using the R language (version 4.1.3; https://www.r-project.org) within RStudio (version 1.4.1106; https://posit.co). The statistical tests were two-sided with an alpha level set at 0.05 unless stated otherwise. The continuous variables were presented as mean ± standard deviation, while categorical variables were summarized as distribution (percentage). The differences among the five databases were assessed by using one-way analysis of variance for continuous variables, and chi-square test or Fisher’s exact test for categorical variables. Inter-reader agreement was evaluated by weighted kappa. The weighted kappa was interpreted as follows: poor (< 0.20), fair (0.20–0.40), moderate (0.40-0.60), good (0.60–0.80), and excellent (≥ 0.80) [35]. The sensitivities, specificities, accuracies, and diagnostic odds ratios were calculated. Subgroup analysis of diagnostic performance was conducted according to (a) gender (male versus female), (b) age (≤ 40 years old versus > 40 years old), (c) data source (local database versus publica database), and (d) T1 signal (T1 much or slightly high signal versus T1 low or high with fluid/fluid level or hemorrhage signal).

## Results

### Patient and lesion characteristics

There were 275 lesions included, including 165 positive and 110 negative lesions (Table 1). The difference in gender, malignancy history, T1WI signal, and T2WI signal was not found among the five databases (all *p* > 0.05), while age (*p* = 0.010), presence of pain (*p* = 0.006), and anatomical site (*p* = 0.008) showed significant difference among the five databases. The detailed characteristics for each patient and lesion are presented in Supplementary Data S1. Representative cases were displayed in Supplementary Figs. S1 to S2.

**Table 1:** Patient and lesion characteristics

	Center A	Center B	Center C	Eurorad	Radiopeadia	Overall	*p*-value
No. of patients	44	50	11	51	119	275	
Age, mean ± standard deviation, median (range)	49.1 ± 18.1	38.1 ± 16.4	37.8 ± 17.2	47.6 ± 19.4	41.1 ± 17.3	42.9 ± 18.0	0.006
Gender, *n* (%)							0.646
Male	20 (45.5)	29 (58.0)	6 (54.5)	31 (60.8)	66 (55.5)	152 (55.3)	
Female	24 (54.5)	29 (42.0)	5 (45.5)	20 (39.2)	53 (44.5)	123 (44.7)	
Malignancy history, *n* (%)^a^							0.102
Present	4 (9.1)	0 (0.0)	0 (0.0)	3 (5.9)	4 (3.4)	11 (4.0)	
Absent	40 (90.9)	50 (100.0)	11 (100.0)	48 (94.1)	115 (96.6)	264 (96.0)	
Pain, *n* (%)							0.006
Present	14 (31.8)	19 (38.0)	7 (63.6)	8 (15.7)	29 (24.4)	77 (28.0)	
Absent	30 (68.2)	31 (62.0)	4 (36.4)	43 (84.3)	90 (75.6)	198 (72.0)	
Anatomical site, *n* (%)							0.008
Upper limb	8 (18.2)	16 (32.0)	3 (27.3)	16 (31.4)	29 (24.4)	72 (26.2)	
Lower limb	32 (72.7)	25 (50.0)	4 (36.4)	18 (35.3)	56 (47.1)	135 (49.1)	
Pelvic bone	2 (4.5)	5 (10.0)	2 (18.2)	9 (17.6)	7 (5.9)	25 (9.1)	
Spine	0 (0.0)	2 (4.0)	2 (18.2)	5 (9.8)	13 (10.9)	22 (8.0)	
Other	2 (4.5)	2 (4.0)	0 (0.0)	3 (5.9)	14 (11.8)	21 (7.6)	
T1 signal, *n* (%)							0.272
Much high	3 (6.8)	5 (10.0)	0 (0.0)	5 (9.8)	16 (13.4)	29 (10.5)	
Slightly high	3 (6.8)	5 (10.0)	1 (9.1)	2 (3.9)	8 (6.7)	19 (6.9)	
Low	38 (86.4)	39 (78.0)	8 (72.7)	42 (82.4)	94 (79.0)	221 (80.4)	
High with fluid/fluid level or hemorrhage	0 (0.0)	1 (2.0)	2 (18.2)	2 (3.9)	1 (0.8)	6 (2.2)	
T2 signal, *n* (%)							0.113
High	37 (84.1)	48 (96.0)	11 (100.0)	49 (96.1)	112 (94.1)	257 (93.5)	
Low	7 (15.9)	2 (4.0)	0 (0.0)	2 (3.9)	7 (5.9)	18 (6.5)	

^a^ Eleven patients with a malignancy history included three lung cancers, three lymphomas, one gastric cancer, one prostate cancer, one renal clear cell carcinoma, one breast cancer, and one malignant tumor of unknown origin

### Inter-reader agreement and diagnostic performance

**Fig. 2:**
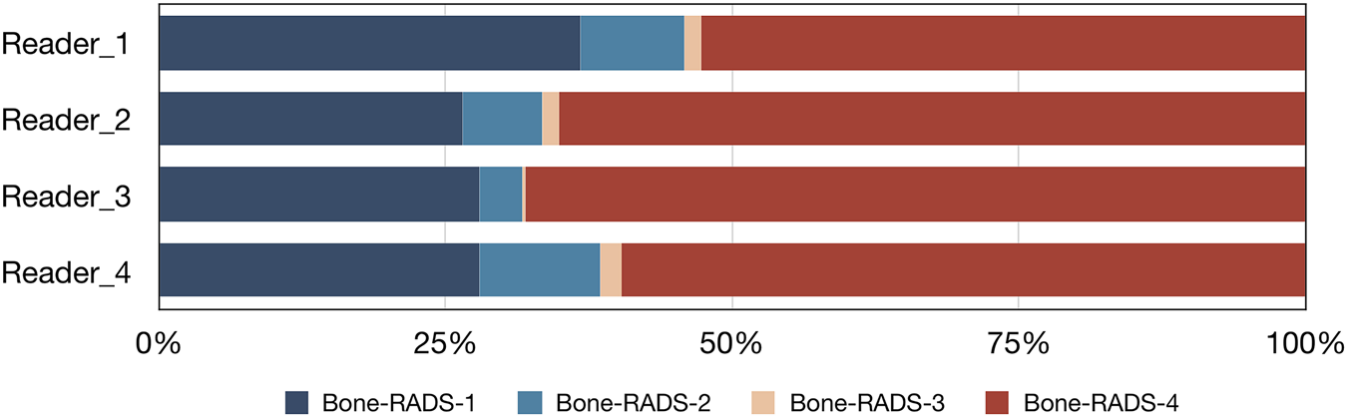
Bar plot of Bone-RADS rating according to readers

**Fig. 3:**
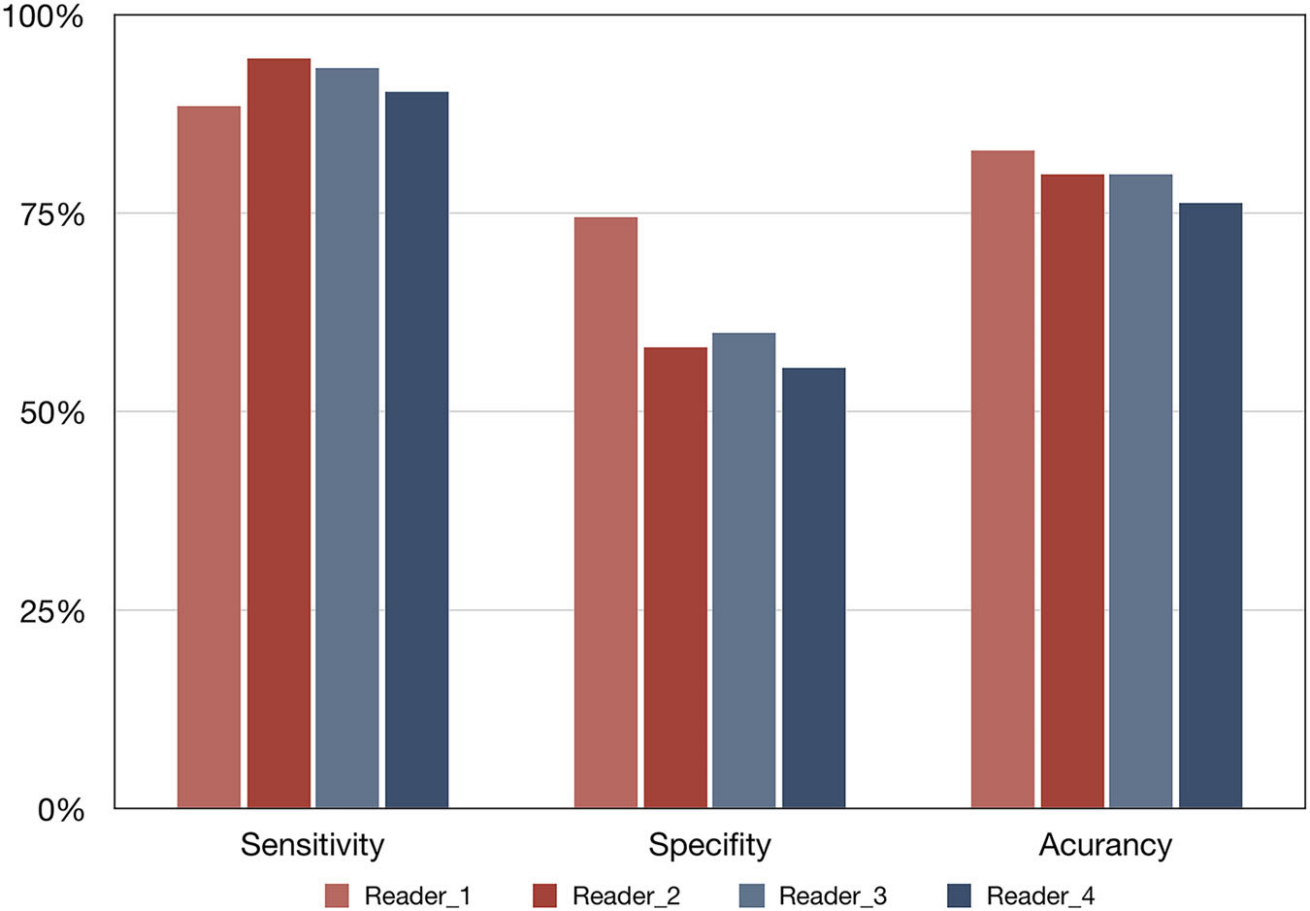
Histogram of diagnostic performance of Bone-RADS according to readers

The 275 included lesions were categorized into Bone-RADS-1 to Bone-RADS-4, respectively (Fig. 2 and Table 2). The inter-reader agreement between two musculoskeletal radiologists and two non-musculoskeletal radiologists was both moderate (weighted kappa of 0.572 and 0.520). The overall inter-reader agreement among four readers was also moderate (weighted kappa of 0.580). The Bone-RADS rating for each lesion per reader is presented in Supplementary Data S2. The diagnostic performance for identifying intermediate or malignant lesions or osteomyelitis ranged according to radiologists with sensitivities of 88.5% to 94.5%, specificities of 55.5% to 74.5%, accuracies of 76.4% to 82.9%, and diagnostic odds ratios of 11.59 to 24.12 (Fig. 3 and Table 3). The subgroup analysis of diagnostic performance is presented in Supplementary Table S1. The diagnostic performance of Bone-RADS-MRI might be better in lesions with T1 signal that is either slightly or much higher, and in those from the local database. The diagnostic performance of Bone-RADS-MRI did not seem to be significantly different between gender and age groups.

**Table 2:** Bone-RADS rating according to readers

	Negative	Positive	Overall
No. of cases	110	165	275
Reader 1			
Bone-RADS-1	81 (73.6)	20 (9.1)	101 (36.7)
Bone-RADS-2	7 (6.4)	18 (1.5)	25 (9.1)
Bone-RADS-3	3 (2.7)	1 (0.6)	4 (1.4)
Bone-RADS-4	19 (17.3)	126 (76.4)	145 (52.7)
Reader 2			
Bone-RADS-1	63 (57.3)	10 (6.1)	73 (26.5)
Bone-RADS-2	12 (10.9)	7 (4.2)	19 (6.9)
Bone-RADS-3	4 (3.6)	0 (0.0)	4 (1.5)
Bone-RADS-4	31 (28.2)	148 (89.7)	179 (65.1)
Reader 3			
Bone-RADS-1	65 (59.1)	12 (7.3)	77 (28.0)
Bone-RADS-2	6 (5.5)	4 (2.4)	10 (3.6)
Bone-RADS-3	1 (0.9)	0 (0.0)	1 (0.4)
Bone-RADS-4	38 (34.5)	149 (90.3)	187 (68.0)
Reader 4			
Bone-RADS-1	60 (54.5)	17 (10.3)	77 (28.0)
Bone-RADS-2	22 (20.0)	7 (4.2)	29 (10.5)
Bone-RADS-3	3 (2.7)	2 (1.2)	5 (1.8)
Bone-RADS-4	25 (22.7)	139 (84.2)	164 (59.6)

*Bone-RADS* Bone Reporting and Data System

**Table 3:** Diagnostic performance of Bone-RADS according to readers

	TP	FP	FN	TN	Sensitivity	Specificity	Accuracy	DOR
Reader 1	146	28	19	82	88.5%	74.5%	82.9%	22.50
Reader 2	156	46	9	64	94.5%	58.2%	80.0%	24.12
Reader 3	154	44	11	66	93.3%	60.0%	80.0%	21.00
Reader 4	149	49	16	61	90.3%	55.5%	76.4%	11.59

*TP* true positive, positive cases that diagnosed as Bone-RADS-2, 3, or 4, *FP* false positive, negative cases that diagnosed as Bone-RADS-2, 3, or 4, *FN* false negative, positive cases that diagnosed as Bone-RADS-1, *TN* true negative, negative cases that diagnosed as Bone-RADS-1, *DOR* diagnostic odds ratio, *Bone-RADS* Bone Reporting and Data System

## Discussion

Our study validated the Bone-RADS-MRI for incidental solitary bone lesions in adults using three local and two public databases. Bone-RADS-MRI achieved moderate inter-reader agreement between musculoskeletal and non-musculoskeletal radiologists, and presented high sensitivity and accuracy but relatively poor specificity.

There is a limited number of validation studies for Bone-RADS [27–30]. Park et al [27] found that Bone-RADS-CT has a good inter-reader agreement, which is better than our study. We suppose that the MRI algorithm is more complex than the CT algorithm, which leads to lower inter-reader agreement in our study. Although we have introduced a training phase before the formal evaluation, the experience and specialty may still contribute to the difference in inter-reader agreement between the two studies. Park et al [27] demonstrated that Bone-RADS-CT has a high sensitivity for lucent malignant lesions but poor specificity and accuracy for both lucent and sclerotic/mixed lesions. Our study also showed high sensitivity and poor specificity, but the accuracy of Bone-RADS-MRI is better than that of Bone-RADS-CT. The imaging modalities of CT and MRI are often combined in the interpretation of bone lesions, while the current Bone-RADS treats them separately. The assessment based only on MRI may have disadvantages in some cases, such as calcification evaluation, but it allows the evaluation of bone lesions when the CT is not available. A head-to-head comparison between Bone-RADS-CT and Bone-RADS-MRI may be necessary to determine whether there is a difference between these two algorithms in diagnostic performance. It is also interesting to find out whether the combination of different imaging modalities can improve the diagnostic performance. However, balancing the cost of imaging and the efficiency of diagnosis would also be important when multiple imaging modalities are recommended. Ramadan et al demonstrated a much higher intra- and inter-reader agreement in Bone-RADS-CT than Park et al [27, 28]. However, our study only showed a moderate agreement. This can be attributed to the readers’ experience and the sample used for assessment. The radiologists in our study are from a general hospital without a musculoskeletal imaging division. Thus, the number of bone lesion cases may not be sufficient for comparison to other centers. Furthermore, our sample may include rarer diseases or uncommon appearances that are worth publishing as case reports but are harder to diagnose correctly. Moreover, the assessment of MRI may be more complex and exhibit more variability in Bone-RADS-MRI. Our study found that the diagnostic performance of Bone-RADS-MRI might be better for lesions with T1 signal that is either slightly or much higher. We suppose that high T1 signal lesions are more characteristic, such as bone marrow, lipoma, hemangioma, intraosseous ganglion, and subchondral cyst. In contrast, the disease spectrum of low T1 signal lesions is more complex, ranging from benign lesions to intermediate and malignant lesions. The readers need more expertise to differentiate low T1 signal lesions from high T1 signal lesions, even with the help of Bone-RADS algorithms.

Although our validation study has confirmed the reproducibility and effectiveness of the Bone-RADS, further comparison with other available RADS is necessary for the full maturity of the Bone-RADS for bone lesions. First, Bone-RADS is developed for only adults. However, there is no clear definition of the age cutoff for the use of Bone-RADS in the white paper. We defined the adults as older than 18 years old in our study, while whether it is appropriate is unclear. BTI-RADS [23] and OT-RADS [24, 25] did not set a limitation of age for the use. We doubt the necessity for emphasizing the concept of adults in Bone-RADS. Further, there is no other version of Bone-RADS in children and teenagers that covers one of the incidence peaks of bone tumors [36]. Second, Bone-RADS is designed for incidental solitary bone lesions. The BTI-RADS also emphasized that the number of lesions should be solitary [23], but other RADS for bone lesions did not set the limitation for the number of lesions [24, 25]. Except for Bone-RADS-CT and Bone-RADS-MRI, other RADS for bone lesions did not restrict the situation for use to incidental findings. We do not consider that Bone-RADS should be limited to incidental solitary lesions, as RADS for other systems were even specialized for specific lesions, and allowed evaluation of multiple lesions [16]. Third, the target user of Bone-RADS was set as non-musculoskeletal radiologists. However, we consider that the system should be further introduced to orthopedists, oncologists, and pathologists to achieve shared concepts and better communications among the stakeholders [11, 17, 18]. Future studies may involve more radiologists and non-radiologists to validate the Bone-RADS to confirm the reliability and generalizability. Fourth, the definition for each Bone-RADS category may need further discussion. The infection is rated as Bone-RADS-4, while the infarct is Bone-RADS-1. It is unclear why Bone-RADS treats these two benign lesions differently. One may argue that the infection needs further treatment, but so do some kinds of infarct [37, 38]. Further, the Bone-RADS-2 of incomplete assessment on imaging is somewhat overlapping with category 0 in other RADS. We believe that the category of Bone-RADS would better follow other RADS [16] and be set according to the malignancy possibility. Fifth, Bone-RADS merged the malignancy possibility of the lesion and the treatment suggestion. This approach may confuse the physicians, especially those who have experience in other RADS. A patient may consider that a Bone-RADS-3 lesion has a higher possibility of malignancy than a Bone-RADS-1 lesion. Other RADS for bone lesions discuss the management suggestions according to the categories and potential histological diagnosis [19, 20, 23–25]. We consider that the suggestion should be peeled from the current version of Bone-RADS. Finally, we consider that RADS for bone lesions should be separated from those for infection [34] and specific disease of myeloma [39]. These two RADS were developed for a specific purpose and should not be mixed up with those for unexpected bone lesions. In addition to these rooms for improvement, the Bone-RADS is still a timely tool for the age of structured reporting [40, 41]. A revised version of Bone-RADS-CT has been proposed [29]. The validation study showed that the revision improves the specificity while keeping the sensitivity. We believe that the Bone-RADS would someday become an efficient system with solid scientific evidence like the ovarian-adnexal RADS (O-RADS) [42]. We are looking forward to developing the system with the skeletal radiology community through a step‐by‐step, structured and systematic approach methodology [43].

There were some limitations to this study. First, our study was conducted using a relatively small sample size with selection bias [30]. The Bone-RADS-1 lesions may have been underreported in center A due to their lesser clinical significance, which resulted in the omission of these cases. The cases from center B were all confirmed by histology, but most of them were excluded due to the unavailability of clinical history. Public databases may prefer to publish cases with typical or unusual radiological appearances. Therefore, the final diagnosis of the included cases did not represent the epidemiological distribution of incidental solitary bone lesions in a general population and potentially influenced our results. Second, a formal evaluation could not be conducted via the picture archiving and communication system, which allows for the evaluation of the whole lesion, as well as adjustments of window level and width. The Bone-RADS rating might have changed in some cases if the evaluation was performed within the settings used in daily practice. However, we believe the current procedure can still be considered as an attempt for a clinical test of Bone-RADS and provides insights for improvements [34]. Third, the reference standard for the diagnostic performance evaluation could have been more robust. Since it is recommended to leave the Bone-RADS-1 lesions alone, most of them were not confirmed by histology but by characteristic features or follow-ups. The reference standard of Bone-RADS rating for public cases was established according to the provided answer, sometimes without detailed supporting information. Fourth, the Bone-RADS-2 and Bone-RADS-3 were not evaluated in our study, as their suitability may change according to the experience and opinion of readers, as well as the attitude and socioeconomic status of the patients. An extra imaging procedure may be necessary for an inexperienced radiologist to reach the correct diagnosis, while follow-up may be preferred by a patient with fibrous dysplasia due to the possibility of malignant transformation [44]. Fifth, our study resulted in a relatively low inter-reader agreement than previous Bone-RADS-CT studies [27, 28]. We supposed that the insufficient experience, sample selection, and imaging modality may have been the potential sources of the disagreement. However, it should be validated in future studies. Sixth, Bone-RADS itself is far from perfect. The Bone-RADS is presented as an easy-to-use decision tree approach, but is still too cumbersome for clinical use. Meanwhile, it can be difficult for non-musculoskeletal radiologists to reach a definitive diagnosis of some Bone-RADS-1 lesions, resulting in a Bone-RADS-4 rating according to the algorithm. Also, the Bone-RADS is currently not known or used by oncologists, orthopedists, or pathologists. Finally, we only retrospectively evaluated the reproducibility and effectiveness of Bone-RADS-MRI. Our study did not investigate whether the Bone-RADS rating had an impact on later imaging work-up or clinical management, let alone the long-term prognosis. Future studies are encouraged to further determine the usefulness of Bone-RADS-MRI for improvements in the education of radiologists, multidisciplinary communication, and eventually patient care.

In conclusion, our study found that Bone-RADS-MRI was of moderate reproducibility and satisfactory effectiveness by four readers using three local and two public databases. Nevertheless, the Bone-RADS-MRI still needs optimization when compared with other available RADS and further clinical validations.

## Supplementary information

ELECTRONIC SUPPLEMENTARY MATERIAL

Supplementary Data

## Data Availability

All data generated or analyzed during this study are included in this published article and its additional files.

## Abbreviations

Bone-RADS: Bone Reporting and Data System
BTI-RADS: Bone Tumor Imaging Reporting and Data System
OT-RADS: Osseous Tumor Reporting and Data System
